# Integrating transcriptomics and metabonomics to unravel modes-of-action of 2,3,7,8-tetrachlorodibenzo-p-dioxin (TCDD) in HepG2 cells

**DOI:** 10.1186/1752-0509-5-139

**Published:** 2011-08-31

**Authors:** Danyel Jennen, Ainhoa Ruiz-Aracama, Christina Magkoufopoulou, Ad Peijnenburg, Arjen Lommen, Joost van Delft, Jos Kleinjans

**Affiliations:** 1 Department of Toxicogenomics, Maastricht University, PO Box 616, 6200 MD Maastricht, the Netherlands; 2 RIKILT-Institute of Food Safety, Wageningen University and Research Centre, PO Box 230, 6700 AE Wageningen, the Netherlands; 3 Netherlands Toxicogenomics Centre, PO Box 616, 6200 MD Maastricht, the Netherlands

## Abstract

**Background:**

The integration of different 'omics' technologies has already been shown in several *in vivo *studies to offer a complementary insight into cellular responses to toxic challenges. Being interested in developing *in vitro *cellular models as alternative to animal-based toxicity assays, we hypothesize that combining transcriptomics and metabonomics data improves the understanding of molecular mechanisms underlying the effects caused by a toxic compound also *in vitro *in human cells. To test this hypothesis, and with the focus on non-genotoxic carcinogenesis as an endpoint of toxicity, in the present study, the human hepatocarcinoma cell line HepG2 was exposed to the well-known environmental carcinogen 2,3,7,8-tetrachlorodibenzo-p-dioxin (TCDD).

**Results:**

Transcriptomics as well as metabonomics analyses demonstrated changes in TCDD-exposed HepG2 in common metabolic processes, e.g. amino acid metabolism, of which some of the changes only being confirmed if both 'omics' were integrated. In particular, this integrated analysis identified unique pathway maps involved in receptor-mediated mechanisms, such as the G-protein coupled receptor protein (GPCR) signaling pathway maps, in which the significantly up-regulated gene son of sevenless 1 (SOS1) seems to play an important role. SOS1 is an activator of several members of the RAS superfamily, a group of small GTPases known for their role in carcinogenesis.

**Conclusions:**

The results presented here were not only comparable with other *in vitro *studies but also with *in vivo *studies. Moreover, new insights on the molecular responses caused by TCDD exposure were gained by the cross-omics analysis.

## Background

Presently, where the current testing strategy for identifying carcinogenic properties of novel chemicals has been designed to effectively capture genotoxic carcinogens, for the identification of important class of non-genotoxic carcinogens no suitable short-term test model, neither *in vivo *nor *in vitro*, is available yet. Till date, discovery of non-genotoxic carcinogenicity is completely depending on the two-year chronic rodent bioassays. These *in vivo *assays not only require a large number of animals, are costly, laborious, and time-consuming, but also cause many false-positive responses and their relevance towards human is questionable. Therefore, alternative assays are needed.

Transcriptomics has been well established in toxicogenomics research [1] and initial studies have indicated that gene expression profiling in both *in vivo *and *in vitro *systems is of value for predicting genotoxicity and carcinogenicity. As the liver is the major target organ for non-genotoxic carcinogens, short term *in vivo *studies have been targeted towards rat liver and these have generated promising gene expression profiles capable of reliably predicting non-genotoxic carcinogenesis [2-4]. Furthermore, promising results have been achieved in our previous studies discriminating genotoxic carcinogens from non-genotoxic carcinogens in the human liver cell line HepG2 [5,6]. The genes identified in these short term *in vivo *and HepG2 studies are involved in processes related to cell cycle, cell signaling, (anti-)apoptosis, oxidative DNA/protein damage response, proliferation, cancer, transcription and protein synthesis. In addition, results of *in vivo *rodent studies, in which a single non-genotoxic carcinogen is examined, e.g. WY-14,643 [7] and 2,3,7,8-tetrachlorodibenzo-p-dioxin (TCDD) [8-10], indicated changes in amino acid metabolism, lipid metabolism and oxidative stress mediated through ligand-activated receptors (e.g. aryl hydrocarbon receptor (AHR), peroxisome proliferator activated receptor (PPAR), pregnane × receptor (PXR; NR1I2), constitutive androstane receptor (CAR; NR1I3)).

Because reported transcriptomic changes induced by TCDD and other non-genotoxic carcinogens in short term *in vivo *studies, referred to the possibility that the endogenous metabolism is affected, we decided to particularly explore the added value of metabonomics analysis in identifying molecular responses *in vitro *caused by non-genotoxic carcinogens. Metabonomics is defined as the study of metabolic responses to drugs, environmental changes and diseases http://www.medterms.com/script/main/art.asp?articlekey=38634. It combines the application of analytical technologies (e.g. nuclear magnetic resonance spectroscopy (NMR), mass spectrometry (MS)) for metabolic profiling with multivariate statistical methods. In general, metabonomics is considered to be complementary to transcriptomics and proteomics [11]. However, this technique shows some distinct features in comparison to these other 'omics' technologies: a) It analyses the end point in a series of changes following exposure to a chemical compound; b) many of the metabolites have a known function; c) changes are detectable intracellularly as well as in extracellular fluids [12]. Various studies illustrate the applications of metabonomics in e.g. toxic risk assessment, biomarker discovery, and studies on toxic mechanisms [13,14], but so far have been performed *in vivo*.

Such *in vivo *studies have also demonstrated that the integration of different 'omics' technologies offers a complementary insight into cellular responses to toxic processes [15-19]. For example, in the study of Coen *et al*. [15] the integration of transcriptomics and metabonomics provided new insight into the toxic consequences for the well-studied therapeutic agent acetaminophen in mice. In adition, Heijne *et al*. [17] showed that the cross-omics analysis provides new insight in the mechanisms of hepatotoxicity in rats exposed to bromobenzene, and also appears to be more sensitive than conventional techniques for indicating induced liver injury. We, therefore, hypothesize that combining of transcriptomics and metabonomics data improves the understanding of molecular responses to non-genotoxic carcinogens also in *in vitro *cellular models, thus potentially contributing to developing cellular assays which are capable of predicting non-genotoxic carcinogenesis *in vivo *[20]. To test this hypothesis, the human hepatocarcinoma cell line HepG2 was exposed to the well-known environmental pollutant TCDD.

TCDD is considered a non-genotoxic human carcinogen (IARC group 1 classification) that activates AHR and induces a broad spectrum of toxic responses including death, immunosuppression, carcinogenicity, and impaired reproduction and development [21,22]. TCDD has a long half-life of 5-10 years in human beings as a result of its high lipophilicity and shows little or no metabolism [23]. The past decades, its effect on the endogenous metabolism *in vivo *as well as *in vitro *has been explored, showing changes in e.g. amino acid metabolism, lipid metabolism and polyamine synthesis [8-10,24-29].

HepG2 was chosen as *in vitro *cell model since it has frequently been applied in toxicogenomics studies [5,6,30-32]. These cells possess a liver-like enzyme pattern [33], expressing many phase I, II and III drug metabolizing enzymes and are competent for regulating these enzymes by ligand-activated transcription factors [34-38]. Furthermore, its metabolizing capacities are evident [39], albeit that metabolic activation does not play a role for TCDD. Furthermore, we have previously observed changes in expression of genes related to the amino acid and lipid metabolism as well as other metabolic processes especially after 48 h of exposure to TCDD in HepG2 [40], which underlines the relevance of this hepatocellular model for the present study.

## Results

### Transcriptomics

The log2 ratios of the filtered data set were used for the selection of significantly modulated DEGs. In total 1,048 DEGs (590 up-regulated; 458 down-regulated) were identified, of which 873 modifications of gene expression levels (505 up-regulated; 369 down-regulated) appeared statistically significant (*p *value < 0.05; False discovery rate (FDR) < 0.08). Expression data of the 1,048 genes are included as additional file 1.

The significantly modulated DEGs were compared with 62 genes affected by TCDD in both rat and mouse hepatic tissue from the studies of Boverhof *et al*. [8] (32 genes) and Boutros *et al*. [41] (33 genes). From these 62 genes 18 were common between the rodent studies and our results. Gene expression changes were in the same direction for 13 common genes, which were mainly involved in (oxidative) stress and xenobiotic responses (e.g. cytochrome P450 (CYP) 1A1, CYP1A2, CYP1B1, NAD(P)H quinone oxidoreductase (NQO1), heme oxygenase (decycling) 1 (HMOX1)).

### Metabonomics

The results of the metabonomics analysis have previously been described by Ruiz-Aracama *et al*. [42] and are summarized below.

#### Analysis of the apolar fraction

The apolar fractions of the cells were first analyzed by ^1^H NMR. From this analysis the main effects in HepG2 cells exposed to TCDD compared to the control were the decrease in the content of triglycerides, cholesterol ester and fatty acids.

GC-MS analysis was also conducted on the apolar fraction of the cells, in order to get better idea of the effect of TCDD on the fatty acid composition of the samples. After exposure to TCDD the level of most fatty acids was decreased. This was even more distinct in the case of shorter chain fatty acids, such as those with 12, 14 and 16 carbon atoms, which were more affected than different isomers of fatty acids of higher chain lengths.

Table 1 shows the final results of the analysis of the apolar fraction.

**Table 1:** Significantly modulated metabolites from the apolar fraction of TCDD-exposed HepG2 (P < 0.01)^a^.

**Fatty acid**^**b**^	**Log2 ratio of TCDD/DMSO**^**c**^
C12:0	-0.32
C14:0	-0.51
C14:1*	-0.51
C14:1*	-1.74
C16:1 (n-6)	-0.74
C16:1 (n-9)	-0.74
C17:0	0.26
C18:0	0.38
C18:1 (n-9)	-0.32
C18:1*	-0.51
C18:2*	-0.51
C18:2*	-0.74
C18:2*	0.38
C18:2*	-0.74
C18:2*	-0.74
C20:1	-0.32
C20:2*	-0.51
C20:2*	-0.51
C20:3*	-0.32
C20:3*	-0.74
C22:2	-0.74
C22:3	-0.51

**Other**	
Triglycerides	-1.00
Cholesteryl ester	-0.51

^a^adapted from Ruiz-Aracama *et al*. [42]

^b^isomers, which cannot be distinguished from each other, are indicated by an asterisk (*)

^c^log2 ratios between TCDD and vehicle control (DMSO).

#### Analysis of the polar fraction

The polar fractions extracted from the exposed cells were first analysed by ^1^H NMR followed by LC-MS. Differences between the polar fraction of the cells exposed to TCDD and those exposed to the control clearly showed a decrease of most amino acids as well as some amino acid conjugates, polyamines and nucleotides. Signal intensities of taurine, citrate, reduced glutathione (GSH), and oxidized glutathione (GSSG), increased with the TCDD treatment. The log2 ratios of all polar metabolites found in this study are shown in Table 2.

**Table 2:** Significantly modulated metabolites from the polar fraction of the TCDD-exposed HepG2 (P < 0.01)^a^.

Metabolite	**Log2 ratio**^**b**^ (^1^H NMR)	**Log2 ratio**^**b**^ (LC-MS)
Leucine, Isoleucine	-0.51	--
Valine	-0.51	--
Alanine	-0.51	--
N-acetylaspartate	-0.51	-0.51
Glutamate	-0.32	--
Glutamine	-0.51	--
Glycine	-0.51	--
Aspartate	-0.51	--
Serine	-0.51	--
Tyrosine	-0.74	-1.32
Proline	--	-1.00
Tryptophan	--	-0.32
Lactate	-1.00	--
Spermidine	--	-2.32
N1-acetyl-spermidine	--	-0.74
Panthotenic acid	--	-0.74
Creatine/Phosphocreatine	-0.51	--
Propyonylcarnitine	--	-0.51
Butyrylcarnitine	--	-0.74
Nucleotides (AMP, ADP, ATP and UMP)	-1.74 to -0.74	--
UMP	--	-0.49
AMP	--	-0.51
Oxidized glutathione	0.38	0.38
Reduced glutathione	0.68	0.26
Taurine	0.49	--
Citrate	0.58	0.77

^a^adapted from Ruiz-Aracama *et al*. [42]

^b^log2 ratios between TCDD and vehicle control (DMSO).

### Pathway analysis and data integration

The significantly modulated DEGs and metabolites were further analyzed with respect to their involvement in different pathway maps. For this purpose, these genes and metabolites were uploaded into MetaCore. In this section significantly modulated maps (*p *value < 0.05 and at least two genes or metabolites present) are further described. FDRs were < 0.4 and < 0.15 for the trancriptomics and metabonomics based maps, respectively. Cellular processes with the corresponding number of maps are listed in Table 3. A complete list of significantly modulated maps per MetaCore analysis can be found as part of additional file 2.

**Table 3:** Cellular processes and pathway maps from MetaCore analyses.

**Input data for MetaCore**^**a**^	Total number of maps	**Cellular process (number of maps)**^**b**^
Transcripts	54	**Amino acid metabolism (14)**
		Carbohydrates metabolism (2)
		**Lipid metabolism (11)**
		**Metabolism of mediators (6)**
		Small GTPase mediated signal transduction (1)
		**Steroid metabolism (9)**
		Transcription (3)
		**Vitamin and cofactor metabolism (5)**
		Other (2)

Metabolites	58	**Amino acid metabolism (21)**
		Apoptosis (2)
		**G-protein coupled receptor protein signaling (10)**
		Immune response (1)
		**Lipid metabolism (5)**
		Nucleotide metabolism (3)
		Transcription (1)
		**Vitamin and cofactor metabolism (4)**
		Other (11)

Integrated data	73	**Amino acid metabolism (21)**
		Apoptosis (1)
		**G-protein coupled receptor protein signaling (14)**
		Immune response (2)
		Intracellular receptor-mediated signaling pathway (2)
		**Lipid metabolism (7)**
		Metabolism of mediators (3)
		Nucleotide metabolism (3)
		Small GTPase mediated signal transduction (1)
		**Transcription (4)**
		**Vitamin and cofactor metabolism (4)**
		Other (11)

^a^The significantly modulated genes, metabolites and integrated data of the TCDD-exposed HepG2 were used as input.

^b^Cellular processes with at least four maps are high-lighted in bold

The most prominent transcriptomics-based maps were involved in metabolic processes with amino acid metabolism on top followed by lipid metabolism, steroid metabolism, metabolism of mediators and vitamin and cofactor metabolism. From the metabonomics-based maps processes involved in metabolism (i.e. amino acid, lipid and vitamin and cofactor metabolism) were most strongly deregulated as wells as G-protein coupled receptor protein (GPCR) signaling.

Comparison between transcriptomics- and metabonomics-based maps reveals an overlap consisting of 14 maps that were mainly involved in amino acid metabolism, but also GSH metabolism (cellular process: Vitamin and cofactor metabolism) was jointly identified by transcriptomics and metabonomics analysis. In additional file 2 these common maps are indicated.

In addition to the analyses of the separate gene and metabolite lists the significant DEGs and metabolites analyzed after 48 h of exposure, were actually combined for the purpose of an integrated analysis of transcriptomics and metabonomics data. This analysis resulted in 164 significantly modulated pathway maps (FDR < 0.23) of which 73 maps contain both significant DEGs as well as significantly modulated metabolites. The cellular processes in which these 73 pathway maps are involved are presented in Table 3.

Cross-omics analysis revealed amino acid metabolism, lipid metabolism, GPCR signaling, Vitamin and cofactor metabolism and transcription to be the cellular processes most dominantly affected in HepG2 by TCDD exposure. For some processes, this was also demonstrated by either transcriptomics or metabonomics analysis (Table 3). Most importantly, the integrated analysis uniquely identified 16 pathway maps which are mainly related to GPCR signaling (9 maps), intracellular receptor-mediated signaling (2 maps) and transcription (2 maps). A complete list of modulated maps retrieved by integrated analysis in MetaCore containing both significant DEGs as well as significantly modulated metabolites is provided as additional file 3.

## Discussion

For integrating read-outs from different 'omics' platforms, i.e. transcriptomics, proteomics and metabonomics, in general, there is no lack of tools that are capable of visualizing data in a biological network context [43]. However, tools that directly integrate e.g. transcriptomics and metabonomics data in a pathway ranking analysis are available only to a limited extent. In this study, MetaCore was used for performing such an integrative analysis based on a combined list of transcriptomics and metabonomics data from TCDD-exposed HepG2 cells. We, thereby, hypothesize that combining of these 'omics' data will improve the understanding of molecular responses caused by TCDD in this *in vitro *heptocellular model. This cross-omics analysis demonstrated specific involvement of pathways related to receptor-mediated processes, cell signaling and endogenous metabolism.

Although different mechanisms may be involved in the carcinogenic action of non-genotoxic compounds, receptor-mediated processes are definitely eligible as possible mechanisms for non-genotoxic carcinogenicity of TCDD [44]. As an AHR-ligand, TCDD induces the transcription of CYP1A1, CYP1B1 and NQO1, which are all significantly up-regulated in this study, and this may lead to the generation of reactive oxygen species (ROS; includes superoxide anion and hydrogen peroxide) and thereby oxidative stress/DNA damage [45-47]. The latter was identified as a specific process for non-genotoxic carcinogenicity [47]. In the study by Knerr *et al*. [45] a slight increase of oxidative DNA damage was measured after incubation of HepG2 cells with TCDD. Furthermore, the increase of GSH and decrease of ATP as found in our study are indicative of oxidative stress. GSH participates in the regulated defense against oxidative stress [48,49], which can be induced by TCDD *in vivo *[46,50,51] as well as *in vitro *[52,53]. ATP was previously shown to be decreased whereas oxidative stress increased upon exposure to TCDD in mice [50,51,54].

**Figure 1:**
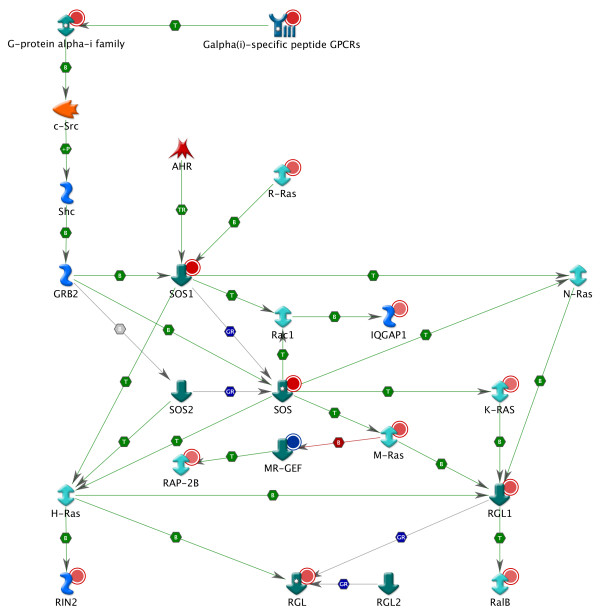
**RAS gene network developed in MetaCore based on available curated data within the tool**. Circles at the top-right of the gene symbols indicate up- (red) and down-regulation (blue) of the genes for TCDD-exposed HepG2. Direct interactions between nodes are shown and include the activation (green), inhibition (red) and unspecified (gray) effects. Detailed information on the symbols can be found at http://www.genego.com/pdf/MC_legend.pdf

Besides the AHR-mediated processes, several GPCR signaling pathway maps as well as other signal transduction pathway maps were significantly modulated in our study (Table 3). These maps include many of the 16 pathway maps uniquely identified by the integrated 'omics' analysis, e.g. "G-Proteins mediated regulation MARK-ERK signaling", "Beta-adrenergic receptors regulation of ERK", "G-Protein alpha-i signaling cascades", "Rap2A regulation pathway" and "PDGF signaling via MAPK cascades". Some of the underlying genes of these pathways belong to the RAS superfamily, a group of small GTPases of which their proteins are activated in a significant fraction of tumors [55,56]. Therefore, these genes could be of importance in the mechanism for non-genotoxic carcinogenicity of TCDD. For example, muscle RAS oncogene homolog (MRAS), related RAS viral (r-ras) oncogene homolog (RRAS), v-Ki-ras2 Kirsten rat sarcoma viral oncogene homolog (KRAS) and v-ral simian leukemia viral oncogene homolog B (RALB) were found to be significantly up-regulated in our study. Members of the RAS superfamily are positively regulated by guanine nucleotide exchange factors (GEFs) [55,56], e.g. son of sevenless (SOS) 1 and ral guanine nucleotide dissociation stimulator-like 1 (RGL1), which are both significantly up-regulated in the present study (Figure 1). GEF-activated RAS interact with downstream effectors, which regulate signaling networks that control gene expression and regulation of cell proliferation, growth arrest, senescence, differentiation, apoptosis and survival [55,56]. As such, SOS1 is important in the cell growth-regulatory MAPK/ERK pathway through the activation of RAS family members [56]. In primary rat hepatocytes SOS has already been shown to be influenced by TCDD [57] while recently, Pierre *et al*. [58] demonstrated that SOS1 is not only controlled by GPCRs, but also by AHR in TCDD-exposed HepG2 (Figure 1). In particular, Pierre *et al*. [58] found an AHR-dependent increase of SOS1 at the mRNA and protein level in TCDD-exposed HepG2.

Other prominent pathway maps significantly affected in HepG2 by TCDD exposure appeared involved in metabolic processes, mainly relating to amino acid and lipid metabolism but also vitamin and cofactor metabolism and metabolism of mediators (Table 3).

**Figure 2:**
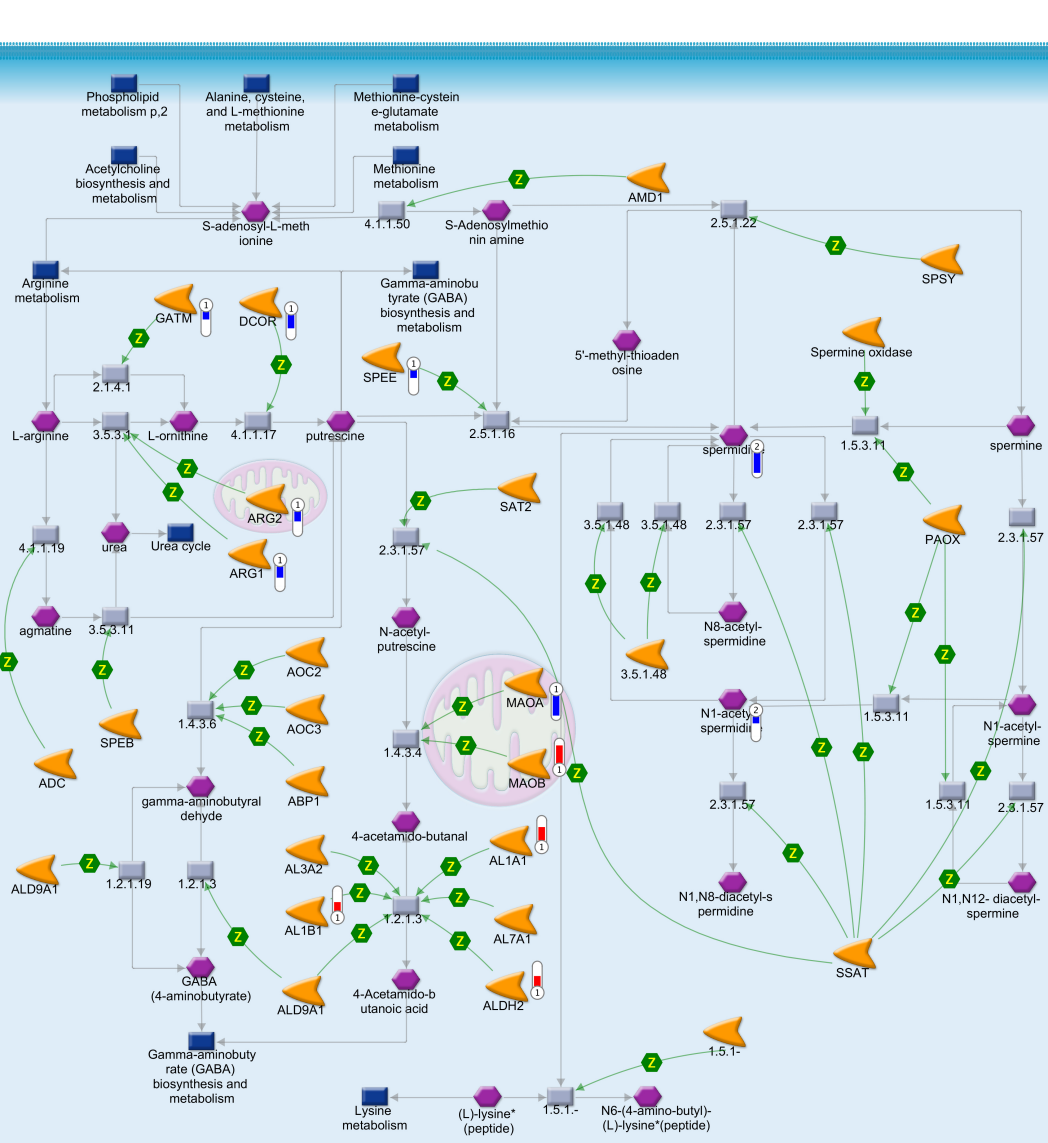
**Polyamine metabolism pathway map in MetaCore including GABA metabolism**. Thermometers indicate up- (red) and down-regulation (blue) of the genes (1) and the metabolites (2) from TCDD-exposed HepG2. Detailed information on the symbols can be found at http://www.genego.com/pdf/MC_legend.pdf

From the set of 20 essential amino acids at least 11 were significantly decreased after exposure to TCDD (Table 2). In addition, many genes involved in the metabolism of these amino acids were down-regulated. For example, glycine and creatine appeared decreased and GATM down-regulated which may affect the synthesis of creatine, an anti-oxidant against ROS, via two reactions [59]. In the first reaction, ornithine and guanidinoacetic acid are formed from glycine and arginine by glycine amidinotransferase (GATM). This reaction is the rate-limiting step in creatine biosynthesis and part of the polyamine metabolism (Figure 2). In the second reaction, catabolyzed by guanidinoacetate N-methyltransferase (GAMT), creatine is produced from guanidinoacetic acid and S-adenosyl-L-methionine.

Another deregulated route involves the contribution of glycine, aspartate and glutamine in the biosynthesis of nucleotides http://themedicalbiochemistrypage.org/nucleotide-metabolism.html. Levels of all three amino acids are decreased upon TCDD-exposure and the gene in the initial step of the purine metabolism, phosphoribosyl pyrophosphate amidotransferase (PPAT) is down-regulated.

**Figure 3:**
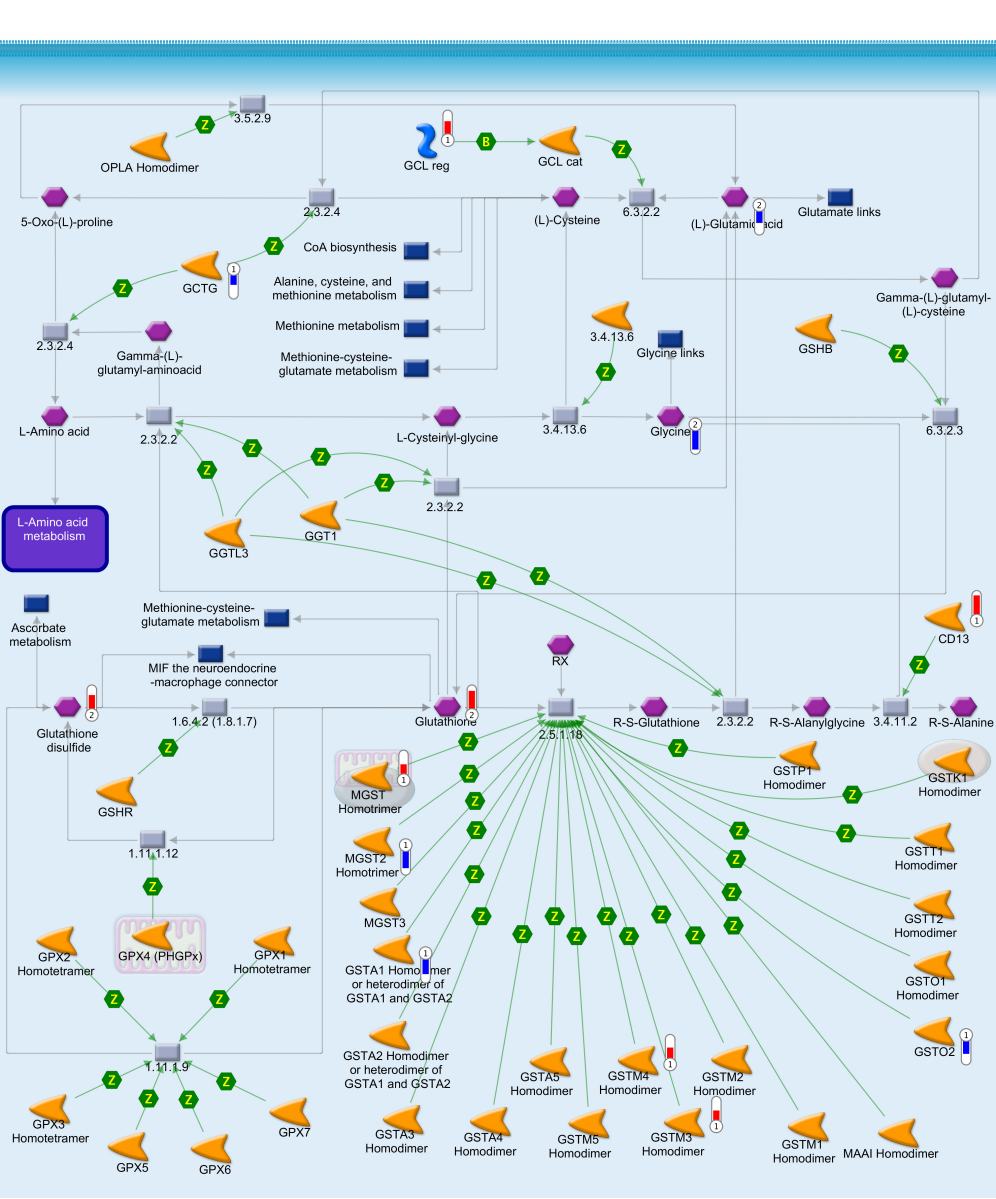
**Glutathione metabolism pathway map in MetaCore**. Thermometers indicate up- (red) and down-regulation (blue) of the genes (1) and the metabolites (2) from TCDD-exposed HepG2. Detailed information on the symbols can be found at http://www.genego.com/pdf/MC_legend.pdf

Furthermore, aminoacyl-tRNA biosynthesis is down-regulated with 12 amino acids decreased and 6 genes down-regulated, thereby most likely inhibiting global protein synthesis. In previous rodent studies [8] it was suggested that genes involved in glutamate, cysteine and glycine metabolism, may be down-regulated to conserve the building blocks of GSH, whereas the genes involved in the synthesis of GSH are up-regulated. Results obtained in our HepG2 model, confirm this suggestion as both GSH and glutathione disulfide (GSSG) are increased upon TCDD-challenge, with a consistent increase in gene-expression of glutamate-cysteine ligase (GCL) which catalyses the production of γ-glutamyl-cysteine, a precursor of GSH (Figure 3).

A further process that is decreased in HepG2 after exposure to TCDD is the production of N-acetylaspartate (NAA) from aspartate and acetyl-CoA. This reaction is catalyzed by aspartate N-acetyltransferase, which recently has been identified as NAT8L [60]. This gene is down-regulated (log2 ratio of -0.83), but not significantly (*p *value = 0.08). NAA is a brain specific protein, but is also found in liver [60,61]. It acts as an acetyl donor for lipid metabolism. For that, NAA is converted to aspartate and acetate. The acetate released in this process, may serve as a precursor in the synthesis of fatty acids by its conversion to acetyl-CoA. The decrease of NAA corresponds well with the decrease of most saturated and unsaturated fatty acids in HepG2 exposed to TCDD. In addition, the gene involved in the first reaction in the fatty acid synthesis from acetyl CoA, i.e. acetyl CoA carboxylase (ACACA), is significantly down-regulated.

Additionally, the sulphur containing amino acid taurine was found to be increased in TCDD-exposed HepG2. Taurine plays an important role in several physiological functions, such as central nervous system neuromodulation, cardiovascular effects, endocrine and metabolic effects and antioxidant/detoxifying activity. It is mainly synthesized in liver and brain, either from cysteine or from methionine, which is first converted to cysteine [62]. However, the genes involved in taurine production, were down-regulated or not changed at all in TCDD-exposed HepG2. Furthermore, taurine is involved in the conjugation of bile acids by bile acid CoA:amino acid N-acyltransferase (BAAT) [63]. The expression of BAAT in this study was down-regulated. These findings indicate that the increase of taurine in HepG2 exposed to TCDD is not the result from an increased taurine production, but rather from a decreased metabolism. The study of Yanagita *et al*. [64] showed that an increase of taurine in HepG2 results in a decrease of triglycerides and cholesteryl ester. In the same study it was suggested that acyl-CoA:cholesterol acyltransferases or sterol O-acyltransferases (SOAT1 and SOAT2), which catalyzes the production of choleteryl ester from cholesterol, is inhibited. The decrease of the triglycerides and cholesteryl ester was also observed in this study and SOAT2 was down-regulated. In another study of taurine-exposed HepG2 [65] microarray data analysis showed taurine-mediated gene expression changes in processes involved in e.g. amino acid metabolism, fatty acid and lipid metabolism, regulation of transcription, immune response, cell cycle, apoptosis and DNA repair. However, from the results of this study it is unclear in what way taurine plays a role in these processes.

From the maps identified by cross-omics analysis to be involved in metabolism of mediators, the polyamine metabolism map also appeared the number 1 ranked pathway map in the transcriptomics gene list and furthermore, is linked to the GABA metabolism [66] (Figure 2). Both polyamine and GABA metabolism have a function in cancer-related processes, whereby an increase of polyamines and/or GABA were observed in various cancer types [67-71]. Our findings suggest that TCDD-exposure in HepG2 decreases polyamine metabolism. In the polyamine metabolism putrescine is the first polyamine produced from which spermidine and subsequently spermine are produced [67,68]. The production of putrescine from ornithine is the rate-limiting reaction. This reaction is catalyzed by ornithine decarboxylase (ODC; DCOR). ODC was significantly down-regulated in this study, as well as arginase 1 and 2 (ARG1, ARG2) or GATM that catalyze the preceding reaction in which ornithine is produced from arginine or from glycine and arginine, respectively. Spermidine and N1-acetylspermidine, an N-acetylated polyamine, were found to be significantly decreased. N1-acetylspermidine is an intermediate for the recycling of spermidine into putrescine [67,68]. These *in vitro *results correspond well with the *in vivo *study of Thomas *et al*. [28], who showed a decrease in the concentration of polyamines in mouse liver after exposure to TCDD. In addition, depletion of polyamines in HepG2 is shown to prevent apoptosis [66]. In our study, exposure of the HepG2 cells to TCDD did not affect apoptosis levels, which were low (< 1%; data not shown).

With regard to the GABA metabolism our results indicated this metabolism to be enhanced. In the GABA metabolism, putrescine is used to form GABA through 4-aminobutanal [66]. The latter reaction is catalyzed by several aldehyde dehydrogenases of which ALDH1A1 (or AL1A1), ALDH1B1 (or AL1B1) and ALDH2 had a significantly up-regulated gene expression after TCDD exposure in HepG2. Furthermore, GABA can be formed from glutamate by glutamate decarboxylase 1 or 2 (GAD1, GAD2), of which GAD1 also has significantly up-regulated gene expression in this study.

## Conclusion

The major result from this study is that TCDD-mediated 'omics' responses in HepG2 are comparable with data from *in vivo *studies on transcriptomics responses induced by non-genotoxic carcinogens in rodent liver. The integrated 'omics' analysis resulted in the identification of unique pathway maps involved in receptor-mediated mechanisms. Such mechanisms have been identified as possible mechanisms for non-genotoxic carcinogenicity *in vivo *[44]; in particular up-regulation of SOS1 seems to play an important role. This resemblance with the *in vivo *situation opens a venue for further developing HepG2 as an *in vitro *model for non-genotoxic carcinogenicity, by applying cross-omics analysis.

Furthermore, integration of transcriptomics and metabonomics data provided novel insights into response pathways to TCDD exposure in HepG2, in particular related to changes in processes involved in amino acid and lipid metabolism as well as glutathione metabolism From these metabolic processes the metabolites taurine, creatine and NAA as well as the genes involved in their metabolism seem to be important for TCDD-mediated carcinogenicity as the elevation of taurine and absence/decrease of creatine and NAA is also observed in human brain and liver tumors [72-76].

Where novel 'omics' technologies such as microRNA analysis and epigenomics, are becoming increasingly available, integrated data analysis seems to represent the way forward in our quest for better understanding human health risks in relation to exposure to toxic agents.

## Methods

### Cell Culture and Treatment

HepG2 cells were obtained from the American Type Culture Collection (Rockville, MD). The cells have a doubling time of about 30 h and grow in clumps. The HepG2 cells were cultured in 6-well plates for transcriptomics in the presence of minimal essential medium (MEM) supplemented with 1% non-essential amino acids, 1% sodium-pyruvate, 2% penicillin/streptomycin and 10% fetal bovine serum (FBS) (all from Gibco BRL, Breda, The Netherlands). For metabonomics HepG2 cells were cultured in T75 flasks using MEM plus Glutamax supplemented with 1% non-essential amino acids, 1% sodium-pyruvate, 1% penicillin/streptomycin and 10% FBS. The cells were incubated at 37°C and 5% CO_2_. When the cells were 80% confluent (density ~1.3 × 10^5 ^cells/cm^2^), the medium was replaced with fresh medium containing either 10 nM TCDD (CAS no. 1746-01-6; Cerilliant, Round Rock, TX, USA), or 0.5% DMSO (Sigma-Aldrich) as a vehicle control. This incubation concentration dose was demonstrated effective in other studies [8,9,40,77,78]. For transcriptomics, treatment was terminated after 48 h by removing the culture medium and immediately adding TRIZOL (Gibco/BRL). For metabonomics, treatment was stopped after 48 h by washing the cells several times with ice-cold 0.9% NaCl and disrupting them by osmotic shock with ice-cold ultra pure water. The cells were harvested using a cell scraper and, together with the leaked metabolites, they were treated ultrasonically to ensure total disruption. Three and four independent biological replicates were conducted for transcriptomics and metabonomics, respectively.

### Transcriptomics

#### Microarray hybridization

Total RNA isolation, target preparation and microarray hybridization of the Affymetrix Human Genome U133 Plus 2.0 GeneChip arrays were performed as described by Jennen *et al*. [40]. The arrays were scanned by means of an Affymetrix GeneArray scanner resulting in. Normalization quality controls, including scaling factors, average intensities, present calls, background intensities, noise, and raw Q values, were within acceptable limits for all chips. Hybridization controls BioB, BioC, BioD, and CreX, were called present on all chips and yielded the expected increases in intensities.

#### Microarray data analyses

A total of six raw data sets was obtained, which were re-annotated to the MBNI Custom CDF-files [79]http://brainarray.mbni.med.umich.edu/Brainarray/Database/CustomCDF/genomic_curated_CDF.asp, RMA normalized [80] and filtered based on the present, marginal and absent calls as previously described by Jennen *et al*. [40]. The resulting filtered data set, consisting of 10,634 unique genes, was used for further analyses.

From the intensities of the filtered data set ratios of treated versus vehicle controls were calculated and subsequently, log2 transformed. Differentially expressed genes (DEGs) were selected using the following criteria: i) an average log2 ratio of < -0.58 or > 0.58 (i.e. absolute fold change of 1.5) for the three replicates, ii) same direction of the log2 ratio for all of three replicates and iii) a log2 ratio of < -0.26 or > 0.26 (i.e. absolute fold change of 1.2) for at least two replicates. Furthermore, significantly modulated DEGs were determined by a paired Student's T-test using *p *value < 0.05 [1]. FDR was determined at this *p *value according to Benjamini and Hochberg [81].

The transcriptomics data in this publication have been deposited in EBI's ArrayExpress http://www.ebi.ac.uk/microarray-as/ae/ and are accessible through ArrayExpress accession number E-MEXP-2817.

### Metabonomics

The metabonomics study was performed on two subcellular fractions, the apolar fraction, containing the apolar metabolites (membranes and intracellular lipids) and the polar fraction, containing the polar and semi-polar intracellular metabolites. Extraction of the metabolites and analysis of the apolar and polar fractions have been described by Ruiz Aracama *et al*. [42]. The apolar fraction was analyzed by means of NMR analysis and GC-MS analysis while on the polar fraction NMR analysis and LC-MS analysis were performed.

#### NMR data analysis

##### Pre-processing of the data and alignment

Visual inspection of the technical replicates (4-fold) of each sample showed a high degree of reproducibility. The NMR data were pre-processed and aligned using an in-house developed program [82].

##### Normalization

The aligned fingerprint data in the form of generated spreadsheets were normalized by using factors obtained from the scaling on the phospholipids signals of the ^1^H NMR spectra of the apolar fraction.

##### Statistical analysis

The normalized spreadsheets were then subjected to statistical analysis using Genemaths XT http://www.applied-maths.com/genemaths/genemaths.htm. Standard analysis entailed performing an ANOVA or Student Test (p < 0.01 after log2 transformation) combined with a PCA. From this type of figure a selection of peak loadings - underlying the separation in the PCA - could be exported. Only differences with a log2 ratio of < -0.26 or > 0.26 (i.e. absolute fold change of 1.2) were taken into account.

#### Identification of metabolites

The identification was done by using commercial standards and/or from literature and databases.

#### MS data analysis

##### Pre-processing of the data and alignment

Visual inspection of the technical replicates (4-fold) of each sample showed a high degree of reproducibility. The GC-MS and LC-MS data are pre-processed and aligned using MetAlign [83]http://www.metalign.nl.

#### Normalization

##### LC-MS dataset

The aligned fingerprint data of the polar fraction dataset, in the form of generated spreadsheets, were normalized by using the phospholipids scaling factors obtained from the ^1^H NMR spectra of the apolar fraction.

##### GC-MS dataset

The identified peaks of GC-MS chromatograms were manually integrated. Normalization of this fraction was done by scaling to the raw values of the integrals of docosahexaenoic and arachidonic methyl esters.

##### Statistical analysis

The normalized spreadsheets of both datasets were subjected to statistical analysis using Genemaths XT. Standard analysis entailed performing a log2 transformation, an ANOVA or Student Test (p < 0.05 or p < 0.01, depending on the fraction) combined with a PCA. From this type of figure a selection of peak loadings - underlying the separation in the PCA - can be exported. Only differences with a log2 ratio of < -0.26 or > 0.26 (i.e. absolute fold change of 1.2) were taken into account.

#### Identification of metabolites

Further analysis and identification was facilitated by using GM2MS, an application of MetAlign that re-creates "new chromatograms" containing only the peaks exported from the PCA selection. Polar metabolites were identified with commercial standards, with FT-MS/MS analysis and using databases. Fatty acids were identified using databases, the eluting order of the peaks and the NIST library.

### Pathway analyses

For the pathway analysis the significant DEGs and metabolites were uploaded into MetaCore (GeneGo, San Diego, CA) for identifying the involvement of these genes or metabolites in specific cellular pathway maps by overrepresentation analyses compared to the total amount of objects involved in the particular maps. As a first step, the transcriptomics and metabonomics data were analyzed separately. In the analysis the filtered data set was used as background list for the transcriptomics data. For the analysis of the metabonomics data no background list could be used. Pathway maps with a *p *value < 0.05 and at least two genes or metabolites present were considered significantly modulated. Within MetaCore FDRs were determined at this *p *value according to Benjamini and Hochberg [81].

As a second step, the individual gene and metabolite lists were combined for an integrated analysis of the transcriptomics and metabonomics responses, i.e. for identifying pathway maps in which both genes as well as metabolites are involved. The pathway analysis with the combined list was done in the same fashion as in the first step. In this analysis no background list could be used and pathway maps with a *p *value < 0.05 and at least two genes or metabolites present were considered significantly modulated. FDR was determined at this *p *value according to Benjamini and Hochberg [81]. From these significantly modified pathway maps, the maps containing both significant DEGs as well as significantly modulated metabolites were further examined.

Finally, the transcriptomics and metabonomics data could be visualized on the pathway maps or on networks created in MetaCore based on the significant pathway maps using the curated information within the pathway tool.

## Competing interests

The authors declare that they have no competing interests.

## Authors' contributions

AP, JK, and JvD participated in the design of the study and overall discussion; CM performed the transcriptomics experiments; AR performed the metabonomics experiments; AR and AL analyzed the metabonomics data; DJ analyzed the transcriptomics data, performed the cross-omics analysis, interpreted the results and drafted the paper. All authors read and approved the final manuscript.

## Supplementary Material

Additional file 1 **DEGs of TCDD-exposed HepG2**. Expression data (log2 ratios) of the 1,048 differentially expressed genes of TCDD-exposed HepG2. Statistical significance (*p *value < 0.05) is indicated. Click here for file

Additional file 2 **Result of MetaCore analyses using transcriptomics and metabonomics data**. A complete list of significantly modulated maps (*p *value < 0.05 and at least two genes or metabolites present) per MetaCore analysis of the TCDD-exposed HepG2. Common maps between the transcriptomics and metabonomics analysis are indicated. Click here for file

Additional file 3 **Result of MetaCore analyses using integrated data**. A complete list of significantly modulated maps (*p *value < 0.05 and at least two genes or metabolites present) retrieved by integrated analysis in MetaCore containing both significant DEGs as well as significantly modulated metabolites. Click here for file
